# State Estimation of an Underwater Markov Chain Maneuvering Target Using Intelligent Computing

**DOI:** 10.3390/e23091124

**Published:** 2021-08-29

**Authors:** Wasiq Ali, Yaan Li, Muhammad Asif Zahoor Raja, Wasim Ullah Khan, Yigang He

**Affiliations:** 1 School of Marine Science and Technology, Northwestern Polytechnical University, Xi’an 710072, China; wasiqali@mail.nwpu.edu.cn (W.A.); liyaan@nwpu.edu.cn (Y.L.); 2 Department of Electrical and Computer Engineering, COMSATS University Islamabad, Attock Campus, Attock 43600, Pakistan; 3 Future Technology Research Center, National Yunlin University of Science and Technology, 123 University Road, Section 3, Yunlin 64002, Taiwan; rajamaz@yuntech.edu.tw; 4 School of Electrical Engineering and Automation, Wuhan University, Wuhan 430072, China

**Keywords:** neural computing, state estimation, Markov chain, turning trajectory, bearings only tracking, maneuvering object

## Abstract

In this study, an application of deep learning-based neural computing is proposed for efficient real-time state estimation of the Markov chain underwater maneuvering object. The designed intelligent strategy is exploiting the strength of nonlinear autoregressive with an exogenous input (NARX) network model, which has the capability for estimating the dynamics of the systems that follow the discrete-time Markov chain. Nonlinear Bayesian filtering techniques are often applied for underwater maneuvering state estimation applications by following state-space methodology. The robustness and precision of NARX neural network are efficiently investigated for accurate state prediction of the passive Markov chain highly maneuvering underwater target. A continuous coordinated turning trajectory of an underwater maneuvering object is modeled for analyzing the performance of the neural computing paradigm. State estimation modeling is developed in the context of bearings only tracking technology in which the efficiency of the NARX neural network is investigated for ideal and complex ocean environments. Real-time position and velocity of maneuvering object are computed for five different cases by varying standard deviations of white Gaussian measured noise. Sufficient Monte Carlo simulation results validate the competence of NARX neural computing over conventional generalized pseudo-Bayesian filtering algorithms like an interacting multiple model extended Kalman filter and an interacting multiple model unscented Kalman filter.

## 1. Introduction

Real-time state estimation of maneuvering object has captured a lot of interest from the research community recently [1]. The phenomena of state prediction found its extensive significance in many practical civilian and defense applications like sonar, radar, indoor tracking, surveillance, unmanned aerial vehicle, smart transportation network, drone navigation, wireless radio network, air traffic control, and bioinformatics [2,3]. Despite its wide range of usage in numerous engineering problems, efficient state prediction still has plenty of room for improvement and various challenges to deal with [4]. Ambiguity in designing the complex maneuvering in state vector of the object and incorrectly measured bearings are the main factors which create complications in state approximation of a maneuvering vehicle [5]. The real-time kinetics of maneuvering targets are often designed by dynamic and measurement models through numerical expression. These mathematical equations of maneuvering targets describe the behavior of motion parameters like speed, velocity, position, course, turning rate and explain how they switch from their present points in real-time [6]. In the case of maneuvering state estimation, a single dynamic model is unable to describe all random maneuvering behaviors of underwater passive vehicles. In the single model, true movement and state vector of the target can be mismatched, and this largely affects the overall performance of the state estimation mechanism [7]. The state prediction models which compute the numerical expressions of target dynamics at each point of trajectory are considered as global and best models [8].

In literature, a lot of valuable research efforts have been done for achieving efficient state prediction of underwater maneuvering objects [9]. A single model Kalman filtering algorithm appeared as the initial attempt in this domain; however, its prediction accuracy is often largely affected by random and complex maneuverings of the underwater object [10]. After that, a conventional full statistic model was proposed by researchers for improving the performance of state approximation in which second-order terms of acceleration are approaching the first-order Markov chain [11]. Although the accuracy of state estimation phenomena improved by a filtering technique based on this model for small maneuverings; meanwhile, the accuracy of this method diverges at constant velocity and high maneuverings of underwater objects. Additionally, the maneuvering process needs more prior statistical information about the target than what usually exists. In the last decade, decision-based algorithms also attracted a lot of attention from the research community which seems better for detecting high maneuverings of the underwater vehicle and handling them efficiently [12]. The variable dimension filter and input estimation algorithms are major examples of this methodology [13,14,15].

The dynamics of the underwater maneuvering target can be efficiently designed with generalized pseudo-Bayesian (GPB) based interacting multiple model (IMM) algorithms, which model the maneuverings of target by using a set of sub-filters [16,17]. However, transition probability information and assumed dynamic models are already known in IMM state estimation techniques [18]. Practically, in GPB algorithms, model switching probabilities are hard to achieve, and it is also difficult to break the dynamics of the target into different multi motion models [19]. Furthermore, computational cost significantly rises with increasing the number of motion models which seriously affects the real-time performance of the overall state estimation scheme. Nevertheless, IMM methodologies have some mutual drawbacks like unnecessary mathematical computations in the case of maneuvering objects and divergence of accuracy because of over motion modeling at maneuvering instants [20]. On the other hand, the maneuvering state estimation problem can be efficiently designed with time series by following state-space modeling [21]. In all the discussed approaches, an independent and identical white Gaussian process noise sequence is considered in the state model of maneuvering objects [22].

Smart computing techniques based on NARX neural networks appeared as an emerging field of study in the last two decades for almost every field of engineering [23,24]. In artificial intelligence computing methodologies, the strength of optimization algorithms and robustness of neural networks are exploited for solving different complex real-life problems [25,26]. Neural intelligence computing schemes are finding their extensive applications in several branches of practical sciences like astrophysics [27], plasma physics [28], atomic physics [29], thermodynamics [30], fluid dynamics [31], electric motors [32], rotating electrical machine [33], electromagnetic [34], meteorology [35], nonlinear optics [36], mathematical equations [37], bioinformatics [38], and nanotechnology [39]. These soft computing schemes performed exceptionally well in the above-mentioned applications and took place of all conventional methodologies [40], while, in correspondence with other traditional techniques, neural networks have produced much better efficiency and convergence rate in all related studies.

The issue of nonlinearity and ambiguity is a major challenge and hard to handle in several real-life practical systems, whose dynamics are time-varying and also depend upon their current state. The NARX neural network paradigm shows a satisfactory convergence rate and can be beneficial for the above-mentioned systems [41]. In literature, time-series data based dynamic inputs are effectively handled with this neural intelligence computing technique [42,43,44]. Application of a neural network for approximating time series is an unsteady approach, where initial knowledge about time series data is not necessary. Maneuvering state prediction of an underwater turning object is typically a nonlinear study in which motion parameters of the target are frequently varied with respect to time.

**Figure 1:**
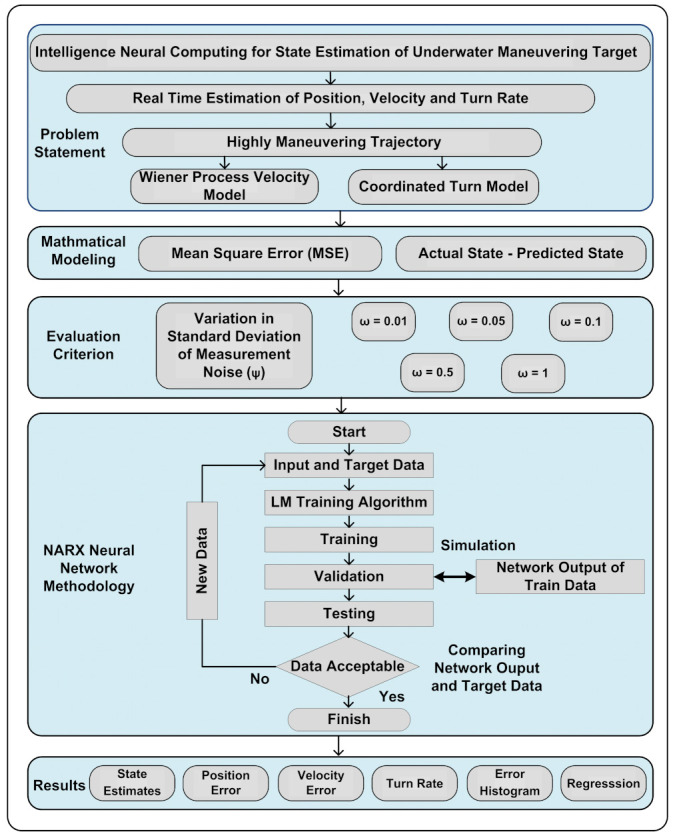
Overall flow chart of the proposed scheme.

A NARX based neural intelligence scheme is mathematically designed in our proposed study for efficiently estimating the real-time state of an underwater maneuvering target in different ocean atmospheres. The time series framework of complex passive bearings from a two-dimensional maneuvering object is comprehensively examined in this work for approximating motion parameters. Different underwater cases for turning and high maneuvering objects are assumed to develop a neurocomputing scheme as a novel application for accurate state prediction in the proposed work. The robustness, strength, and precision of neural intelligence computing are equated with our recently recorded work [16], in which a similar state approximation model is investigated with GPB based algorithms like an interacting multiple model extended Kalman filter (IMMEKF) and an interacting multiple model unscented Kalman filter (IMMUKF). Probabilistic variations of measured noise in the Gaussian environment are used to design ideal and cluttered undersea scenarios to compare the performance of the proposed scheme with conventional filtering methodologies. Position, velocity, and turn rate based state estimation is conducted in this work by exploiting the potency of NARX neurocomputing strategy. A detailed and briefed graphical abstract of the proposed study is shown in Figure 1. The salient features of the conducted study are outlined as:

(1) The strength and potency of the NARX based neurocomputing paradigm are extensively investigated for accurate state approximation of a maneuvering underwater Markov chain target.
(2) State estimates, position error, velocity error, turn rate estimates, error histogram, and regression analysis of NARX are computed for passive turning targets and compared with conventional nonlinear and multiple model variants of the Kalman filter like IMMEKF and IMMUKF.
(3) Motion features of the kinetic target in the highly maneuvering trajectory are designed by utilizing the firmness of the well-known Wiener process velocity (WPV) model and the coordinated turn (CT) model.
(4) The standard deviation of measurement noise is selected as an evaluation criterion, and its numerical values are varied in simulations for analyzing the trend of given techniques.
(5) All the given algorithms are compared on the basis of a minimum mean square error (MSE), which is chosen as the performance matrix and simulation outcomes show that the accuracy of the NARX based neural network is far better from multiple model Kalman filters for estimating the real-time state of an underwater maneuvering object.

The rest of the study is organized in the following pattern. Section 2 provides bearings only tracking (BOT) based multiple model maneuvering state estimation system design in two-dimensional Cartesian coordinates. Comprehensive mathematical modeling of continuously turning object is also developed in this section. The structure and working principle of the NARX neural intelligence network are explained in Section 3 with the process of training, testing, and validation. Simulation outcomes and discussion in the context of minimum MSE of given methods are described in Section 4. The last section of the proposed work reveals the major contributions and defines further goals for future research.

## 2. Markov Chain Maneuvering State Estimation System Model

**Figure 2:**
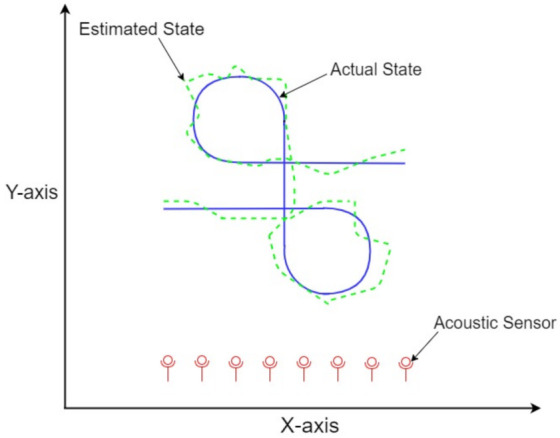
Maneuvering state estimation framework.

A rectangular coordinates’ two-dimensional state prediction system modeling of the Markov chain maneuvering target is mathematically designed in this section of the study. The complex ocean medium is assumed in this scheme for precise real-time state prediction of a continuous turning object by deploying state space-based BOT phenomena. Passive noisy bearings from the maneuvering object at each time instant are collected from eight acoustic sensors, and these sensors are regarded as observer platforms. The sensors are deployed along a line to involve the idea of Uniform Linear Array (ULA) and to obtain maximum benefits of ULA in maneuvering target tracking. This nonlinear complex data collected at observers is called the bearing of the moving object, and it is largely based on the position of each sensor. The arrangement of observers is done in such a manner that there is an equal distance between each observer. A continuous turning trajectory of the underwater target is assumed in this proposed Markov chain maneuvering state estimation system, which is being estimated by an intelligence neural computing scheme. The movements of maneuvering object and estimation architecture are illustrated in Figure 2.

The modeling parameters of many practical systems fluctuate with respect to time. One particular system model is unable to define these varying system parameters. In real-time state estimation problems, there is always the probability that modeling parameters might change during the prediction process. These types of systems are referred to as Markov chain or multiple model systems. In these scenarios, the selection of a single system model may lead to divergence of the overall scheme. Consequently, it is necessary to design a generic state model of a moving object which can have the properties of different system models. Here, in this study, WPV and CT models are applied for defining the dynamics of the underwater maneuvering target.

### 2.1. Wiener Process Velocity (WPV) Model

The instantaneous state of the maneuvering object at time step t with position $\left(x_{t},y_{t}\right)$ and velocity $\left({\dot{x}}_{t},{\dot{y}}_{t}\right)$ in two-dimensional Cartesian coordinates is shown in state vector $Y_{t}^{a}$ as:

(1) $$Y_{t}^{a}={\left[\begin{array}{cccc} x_{t}^{a} & y_{t}^{a} & {\dot{x}}_{t}^{a} & {\dot{y}}_{t}^{a} \end{array}\right]}^{T}.$$

In the meantime, the state vector at the receiving station in Cartesian coordinates can be defined as:

(2) $$Y_{t}^{b}={\left[\begin{array}{cccc} x_{t}^{b} & y_{t}^{b} & {\dot{x}}_{t}^{b} & {\dot{y}}_{t}^{b} \end{array}\right]}^{T}.$$

The respective state vector of the maneuvering object and base station is given as:

(3) $$Y_{t}=Y_{t}^{a}-Y_{t}^{b}={\left[\begin{array}{cccc} x_{t} & y_{t} & {\dot{x}}_{t} & {\dot{y}}_{t} \end{array}\right]}^{T}.$$

Dynamics of maneuvering object are designed according to the discrete-time WPV model in the domain of state-space phenomena. According to this model, the state equation can be modeled as:

(4) $$Y_{t+1}=A_{t}Y_{t}+\xi_{t}^{P}.$$

In the above given state equation, the state transition matrix is represented with $A_{t}$ having dimensions of i×i. In the state-space methodology, the state transition matrix describes the feedback of the dynamic model, while process noise $\xi_{t}^{P}$ is assumed in Gaussian distribution with zero mean. The distribution of state transition matrix $A_{t}$ with sampling space Δt is assumed as follows:

(5) $$A_{t}=\left[\begin{array}{cccc} 1 & 0 & \Delta t & 0\\ 0 & 1 & 0 & \Delta t\\ 0 & 0 & 1 & 0\\ 0 & 0 & 0 & 1 \end{array}\right],$$

and this sampling space Δt is defined as:

(6) Δt=[(t+1)−t].

The state pace model explained in Equation (4) must be written in discrete time for precise prediction of motion parameters with the NARX scheme. The reason for choosing a discrete-time kinetic model is that this modeling can better evaluate the performance of the system for time instant t, which is a multiple of sampling space Δt. The updated form of state equation in discrete time with its appropriate parameters is given as:

(7) $$Y_{t+1}=\underset{A_{t}}{\underset{︸}{\left[\begin{array}{cccc} 1 & 0 & \Delta t & 0\\ 0 & 1 & 0 & \Delta t\\ 0 & 0 & 1 & 0\\ 0 & 0 & 0 & 1 \end{array}\right]}}\underset{Y_{t}}{\underset{︸}{\left[\begin{array}{c} x_{t}\\ y_{t}\\ {\dot{x}}_{t}\\ {\dot{y}}_{t} \end{array}\right]}}+\xi_{t}^{P}.$$

The Gaussian distributed process noise $\xi_{t}^{P}$ can be modeled with covariance $B_{t}$ as:

(8) $$\xi_{t}^{P}\approx {N(0,B}_{t}).$$

For achieving relatively slow turns of the object’s trajectory, the value of process variance is taken 0.05 in simulations.

### 2.2. Coordinated Turn (CT) Model

A specific dynamic model that is commonly used for defining the motion parameters of the maneuvering target, which is frequently moving in a turning track is known as the CT model. In this dynamic modeling, the state vector holds a supplementary parameter termed as the turn rate. For the CT model, the state vector of the maneuvering object in Cartesian coordinates for its position, velocity and turn rate can be written as:

(9) $$Y_{t}^{a}={\left[\begin{array}{ccccc} x_{t}^{a} & y_{t}^{a} & {\dot{x}}_{t}^{a} & {\dot{y}}_{{}_{t}}^{a} & \varphi_{t}^{a} \end{array}\right]}^{T}.$$

In addition, the state vector at the observer platform can be designed as:

(10) $$Y_{t}^{b}={\left[\begin{array}{ccccc} x_{t}^{b} & y_{t}^{b} & {\dot{x}}_{t}^{b} & {\dot{y}}_{{}_{t}}^{b} & \varphi_{t}^{b} \end{array}\right]}^{T}.$$

Meanwhile, a corresponding state vector among the maneuvering object and observer platform is given as:

(11) $$Y_{t}=Y_{t}^{a}-Y_{t}^{b}={\left[\begin{array}{ccccc} x_{t} & y_{t} & {\dot{x}}_{t} & {\dot{y}}_{t} & \varphi_{t} \end{array}\right]}^{T}.$$

The state equation in the CT model is defined as:

(12) $$Y_{t+1}=A_{t}Y_{t}+\mu \xi_{t}^{P}.$$

The above state equation in the CT model is almost identical as in the WPV model given in (4) with an extra parameter, which actually explains the transfer function of Gaussian distributed process noise. Likewise, the WPV model, the discrete form of the state equation for the CT model is illustrated below as:

(13) $$Y_{t+1}=\underset{A_{t}}{\underset{︸}{\left[\begin{array}{ccccc} 1 & 0 & \frac{\sin(\varphi_{t}\Delta t)}{\varphi_{t}} & \frac{\cos(\varphi_{t}\Delta t)-1}{\varphi_{t}} & 0\\ 0 & 1 & \frac{1-\cos(\varphi_{t}\Delta t)}{\varphi_{t}} & \frac{\sin(\varphi_{t}\Delta t)}{\varphi_{t}} & 0\\ 0 & 0 & \cos(\varphi_{t}\Delta t) & -\sin(\varphi_{t}\Delta t) & 0\\ 0 & 0 & \sin(\varphi_{t}\Delta t) & \cos(\varphi_{t}\Delta t) & 0\\ 0 & 0 & 0 & 0 & 1 \end{array}\right]}}Y_{t}+\underset{\mu }{\underset{︸}{\left[\begin{array}{c} 0\\ 0\\ 0\\ 0\\ 1 \end{array}\right]}}\xi_{t}^{P}.$$

In this dynamic modeling, white Gaussian noise is also assumed with zero mean, and its covariance is similar as that given in Equation (8):

(14) $$\xi_{t}^{P}\approx N(0,B_{t}).$$

The mathematical computations of the CT model are given in matrix form while its behavior is nonlinear. Thus, the CT model can be designed with five mathematical expressions as:

(15) $$x_{t+1}=x_{t}+\frac{\sin(\varphi_{t}\Delta t)}{\varphi_{t}}{\dot{x}}_{t}+\frac{\cos(\varphi_{t}\Delta t)-\Delta t}{\varphi_{t}}{\dot{y}}_{t},$$

(16) $$y_{t+1}=y_{t}+\frac{1-\cos(\varphi_{t}\Delta t)}{\varphi_{t}}{\dot{x}}_{t}+\frac{\sin(\varphi_{t}\Delta t)}{\varphi_{t}}{\dot{y}}_{t},$$

(17) $${\dot{x}}_{t+1}=\cos\left(\varphi_{t}\Delta t\right){\dot{x}}_{t}-\sin\left(\varphi_{t}\Delta t\right){\dot{y}}_{t},$$

(18) $${\dot{y}}_{t+1}=\sin\left(\varphi_{t}\Delta t\right){\dot{x}}_{t}+\cos\left(\varphi_{t}\Delta t\right){\dot{y}}_{t},$$

(19) $$\varphi_{t+1}=\varphi_{t}+\xi_{t}^{P}.$$

The variance of Gaussian process noise $\xi_{t}^{P}$ for turn rate $\varphi_{t}$ is set to $Y_{\varphi }$ = 0.15 in simulations for assuming relatively high maneuverings.

### 2.3. Measurement Model

In this state estimation modeling, both WPV and CT models have the same measurement model, which is also designed with the principle of state-space phenomena. The mathematical expression of the measurement model at time step t is given as:

(20) $$X_{t+1}=H\left(Y_{t+1},\xi_{t+1}^{M}\right).$$

Real-time passive maneuverings from the turning object at time step t are integrated in a H(.) matrix, which is also known as measurement function. It contains complex measurements by following the methodology of the point–slope tangent relationship. In the above measurement model, independent Gaussian distributed measurement noise is denoted with ξ at time step t. Passive measurements at sensors are obtained with the real position of a dynamic target and orientation of sensors by using the following relationship as:

(21) $$H\left(Y_{t+1}\right)=\underset{\mathrm{bearings}}{\underset{︸}{\mathrm{arctangent}\left[\frac{y_{t}-\Lambda_{y}^{e}}{x_{t}-\Lambda_{x}^{e}}\right]}}.$$

In two-dimensional Cartesian coordinates, the real-time position of maneuvering target is represented with $\left(x_{t},y_{t}\right)$ while localization of sensors *e* is denoted with $\left(\Lambda_{x}^{e},\Lambda_{y}^{e}\right)$ in the above measurement function. Measurement model X given in (20) can be written in an updated form for acoustic sensors *e* at time step t as:

(22) $$X_{t}^{e}=\mathrm{arctangent}\left[\frac{y_{t}-\Lambda_{y}^{e}}{x_{t}-\Lambda_{x}^{e}}\right]+{\xi_{t}^{e}}^{M}.$$

Measurement noise ${\xi_{t}^{e}}^{M}$ in the above relationship has zero mean, while its covariance T can be computed as:

(23) $${\xi_{t}^{e}}^{M}\approx N\left(0,T_{t}\right),$$

(24) $$T_{t}=diag(\omega_{X}^{2}).$$

In Equation (24), the standard deviation of measurement noise is denoted with $\omega_{X}$, which plays a vital role in analyzing the accuracy of state estimation techniques in the maneuvering target tracking framework. Variation of the standard deviation of measurement noise defines the behavior of the undersea atmosphere. In our study, different values of the standard deviation of measurement noise are chosen for investigating the convergence and robustness of the neural intelligence scheme and GPB algorithms. The coordinated turning trajectory of the underwater object is developed by following these steps.

(1) Initial location of maneuvering object is the origin (0, 0), having a constant velocity of 1 towards *x*-axis e.g., $\left({\dot{x}}_{t},{\dot{y}}_{t}\right)$ = (1, 0).
(2) A right turn is taken by target after 4 s of starting point with turn rate $\varphi_{t}$ = −1.
(3) The target ends turning right at the total time of 9 s and moves straight with a constant velocity of 1 for the next 2 s.
(4) At the total time of 11 s, the underwater target maneuver for the left turn with turning parameter $\varphi_{t}$ = 1.
(5) At the total time of 16 s, the target ends turning on the left side and moves straight with a similar velocity for 4 s.

## 3. Intelligent Neural Computing

In this section, we designed an NARX neural intelligence mathematical model for the efficient state prediction of the kinetic target in an underwater medium. In neural computing, synchronization between supplementary external information and designed time series cannot be ignored. In the problems of parameter estimation, the behavior of the applied method is associated with noise depraved bearing. Hence, the information of measurement noise or preceding data are usually incorporated for time series modeling to find the satisfactory accuracy.

### 3.1. Nonlinear Autoregressive with the Exogenous Input (NARX) Neural Scheme

For the mathematical modeling of NARX neural intelligent computing, supervised learning of neurons is used for finding the next information in time series by precisely integrating preceding values. In time-series data, the external input and second output are responsible for estimating future values in the designed structure of the NARX neural computing. Therefore, input, output, delay, and hidden layers are combined for designing a multilayer architecture of the NARX model. The time series of state equation Y(t) is estimated for *n* preceding points of actual state Y with external input series of measurements X(t) for a maneuvering object using the delay factor of *m*. For estimating required time series, hidden nodes of NARX neural scheme are designed as:

(25) $$Y\left(t\right)=k\left(\begin{array}{c} X(t-1),\;X(t-2),\;\cdots ,\;X(t-m)\\ Y(t-1),\;Y(t-2),\;\cdots ,\;Y(t-n) \end{array}\right)+\eta \left(t\right).$$

In the above designed nonlinear model of NARX neural intelligence learning, measurements X(t) is a *j*-dimensional additional time series while Y is denoting output time series with dimensions of *k*. The error of neural structure is η(t), while output delays are represented with *m* and *n*, respectively:

(26) $$q\left(t\right)=f_{\rho_{1}}\left(X\left(t\right)w_{\rho_{1}}+\gamma_{\rho_{1}}\right),$$

(27) $$Y\left(t\right)=f_{\rho_{2}}\left(q\left(t\right)w_{\rho_{2}}+\gamma_{\rho_{2}}\right).$$

**Figure 3:**
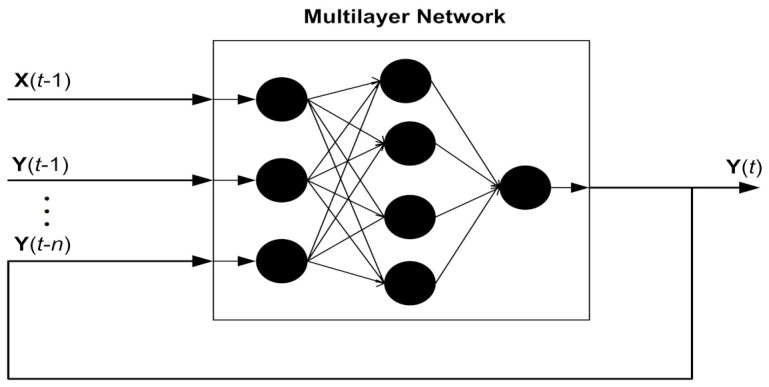
The multilayer structure of the NARX neural network.

In the above equations, the hidden layer vector is denoted with q(t) having dimensions of ρ. Weights between multilayers are represented by $w_{\rho_{1}}$ and $w_{\rho_{2}}$ correspondingly. However, thresholds of hidden layers and input layers are represented with $\gamma_{\rho_{1}}$ and $\gamma_{\rho_{2}}$ accordingly. The network function of hidden nodes is $f_{\rho_{1}}$, and the activation response of output nodes is shown by $f_{\rho_{2}}$. A multiple layer paradigm of NARX technique is depicted in Figure 3.

### 3.2. Architecture of an NARX Neural Scheme

It is important to note that NARX is a recursive discrete-time neural network, which consists of a time series and can be designed as:

(28) $$Y\left(t+1\right)=k\left(\begin{array}{c} Y(t),\;Y(t-1),\;\cdots ,\;Y((t-c)+1)\\ X(t-(n+1)),\;\cdots ,\;X((t-c)-(n+1)) \end{array}\right).$$

The time delay parameter n is assumed null in the above equation. Based upon this assumption, the updated form of NARX model is shown as:

(29) $$Y\left(t+1\right)=k\left(\begin{array}{c} Y(t),\;Y(t-1),\;\cdots ,\;Y(t-c,+1)\\ X(t),X(t+1),\;\cdots ,\;X(t-c,+1) \end{array}\right).$$

The above function is expressed in vector form as:

(30) Y(t+1)=k(Y(t),X(t)).

**Figure 4:**
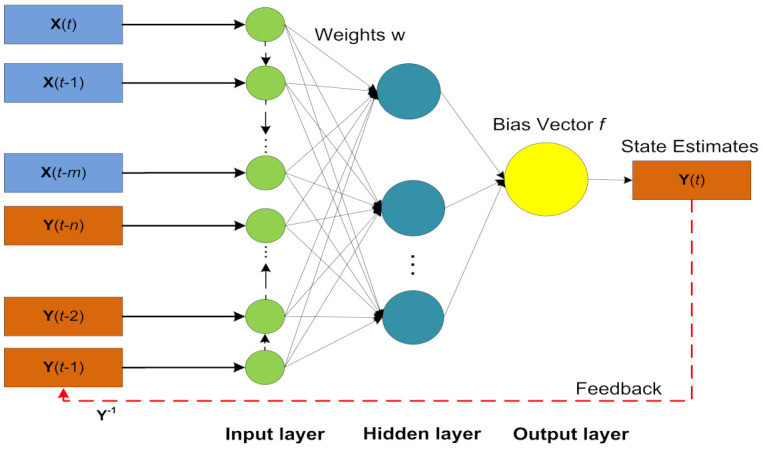
Architecture of the NARX neural network.

In the above vector form, input and output regress are represented with vectors X(t) and Y(t) correspondingly. For the efficient training of the NARX neural model, we applied a Levenberg–Marquardt (LM) based algorithm. The detailed design of NARX neural strategy is represented in Figure 4.

### 3.3. Levenberg–Marquardt (LM) Training Method

The LM method is a well-known training setup found in literature for designing an NARX neural network. It is also known as the fastest learning mechanism for the formulation of an algorithm. Its training efficiency is far better from other training algorithms because it computes the second-order derivatives without solving the Hessian matrix. The two scientists Donald Marquardt and Kenneth Levenberg introduced the concept of the LM training method. Minima of measurement function J(X) is calculated initially in the LM technique with the summation of squares for nonlinear functions as:

(31) $$J\left(X\right)=\sum_{\alpha =1}^{\beta }\frac{1}{2}{\left[j_{\alpha }\left(X\right)\right]}^{2}.$$

The Hessian function P and gradient parameter *g* are computed as:

(32) $$P=S_{t}^{T}S_{t},$$

(33) $$g=S_{t}^{T}\eta_{t}.$$

In the above Hessian matrix and gradient equation, S is representing the Jacobian matrix. Here, we combined the first-order derivatives of neural network error in correspondence with weights and biases, whereas the overall error of the NARX neural model for all training samples is shown with $\eta_{t}$. The Jacobian matrix is only used for necessary computations in the LM training scheme. However, the means of the squared errors are combined in the performance function. The search space for the LM training technique is calculated in the following order as:

(34) $$\left(S_{t}^{T}S_{t}+{℘}_{t}I\right)\vartheta_{t}=-S_{t}^{T}j_{t}.$$

In the given search space function, an identity matrix is represented with I and positive scalars are denoted with ${℘}_{t}$ giving increment of $\vartheta_{t}$. Therefore, the principle of updating weights $w_{t}$ in the training mechanism is expressed as:

(35) $$w_{t+1}=w_{t}-\frac{S_{t}\eta_{t}}{\left(S_{t}^{T}S_{t}+{℘}_{t}I\right)}.$$

### 3.4. Performance Evaluation Criterion

At every time instant t, the least mean square position error between a true and approximated position for turning object is formulated as an evaluation criterion for neural intelligence computing methodology. In this study, it shows the precision and convergence for a neural intelligence technique. On the other hand, the MSE function of NARX, IMMEKF, and IMMUKF are formulated for each independent Monte Carlo simulation as:

(36) $$\mathrm{MSE}\left(t\right)=\frac{1}{N}\sum_{t=1}^{N}{∥Y_{t}^{True}-Y_{t}^{Est}∥}^{2}$$

In the above MSE function, we denote the true state for turning objects as $Y_{t}^{True}$, while an approximated state of the target with NARX and filtering techniques is represented by $Y_{t}^{Est}$. However, the total number of data points is shown by *N* which are 200 in simulations, whereas t = 1 is the initial data point. These position errors are computed at each time step t for the turning trajectory.

## 4. Simulation Results and Discussion

In this section of the study, simulation results in the sense of real-time state approximates, position error, velocity error, turn rate estimates, error histogram, and regression are briefly explained for proposed NARX based neural computing.

The standard deviation of measured Gaussian noise is chosen as a performance parameter and numerically varied from 0.01 to 1 radian in simulations for designing five different cases. The highest value of measured noise ω = 1 radian is showing a cluttered ocean environment, while minimum value ω = 0.01 radian depicts the ideal atmosphere. In simulations, there are many mathematical variables in state estimation phenomena that need to be accurately adjusted for acquiring the required performance. Suitable values of state estimation parameters are given in Table 1.

**Figure 5:**
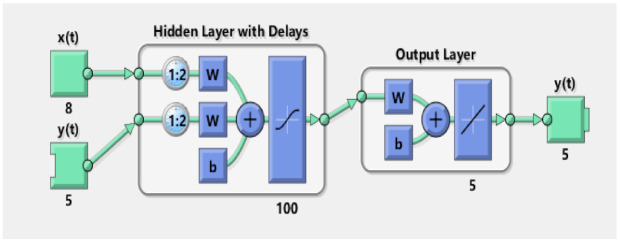
Architecture of the NARX neural network.

Passive bearings combined at acoustic observers are denoted with X(t) and the actual state of maneuvering target represented with Y(t) are applied to NARX neural network as inputs for computing approximated state Y(t). The neural network toolbox for NARX in the MATLAB software package is used for the conduction of the simulations. The toolbox structure of NARX Model is based on input, hidden, and output layer as shown in Figure 5.

The actual state vector in Equation (11), which consists of five entities like *x*–*y* positions, *x*–*y* velocities, and the turning parameter is applied on one input while passive measurements from eight sensors are applied as another input to the NARX model for obtaining the estimated state vector of five elements. In simulations, 100 hidden neurons are used in the hidden layer while a sigmoid function is used for activation of these neurons. The process of training weights for NARX is done with the LM method by using backpropagation through time methodology. The epoch mode strategy is conducted in the training process of the neural network. In the designing of the NARX neural network, 70% of the data of the complete time series is used for the training process, while the remaining 30% is divided equally in the validation and testing procedure of the obtained results.

### 4.1. State Estimation Analysis of Markov Chain Maneuvering Target for Different Cases of the Standard Deviation of Measurement Noise

**Figure 6:**
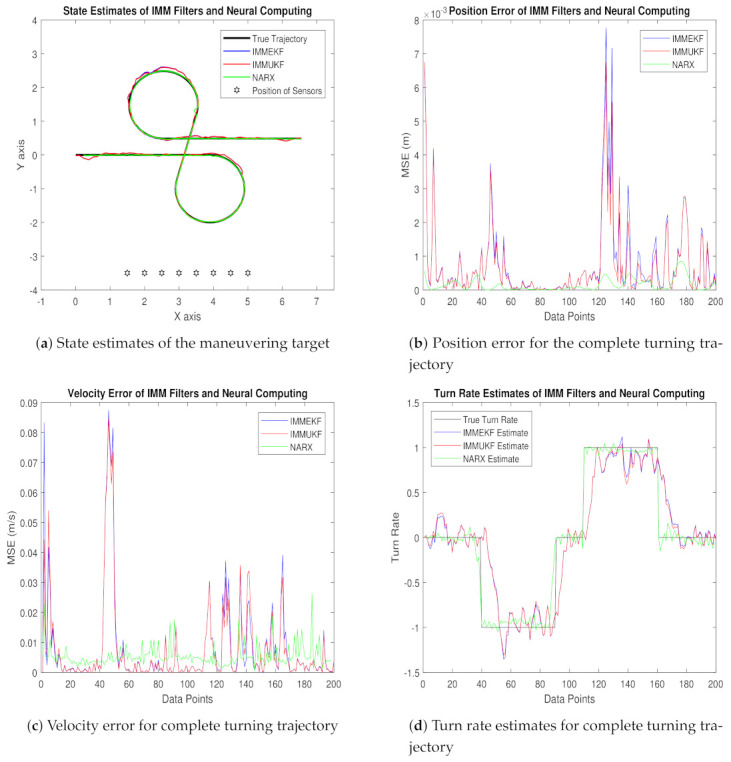

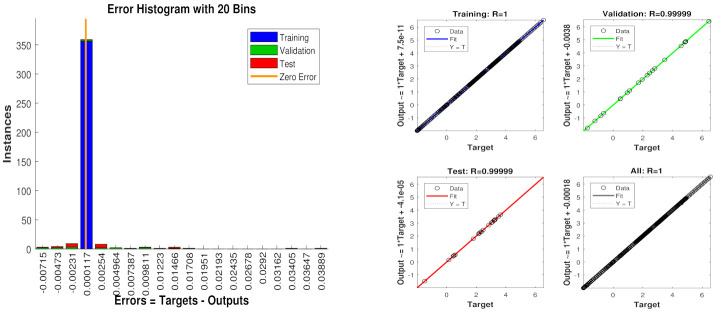
State estimation performance of IMMEKF, IMMUKF, and NARX for ω = 0.01 radian.

**Figure 7:**
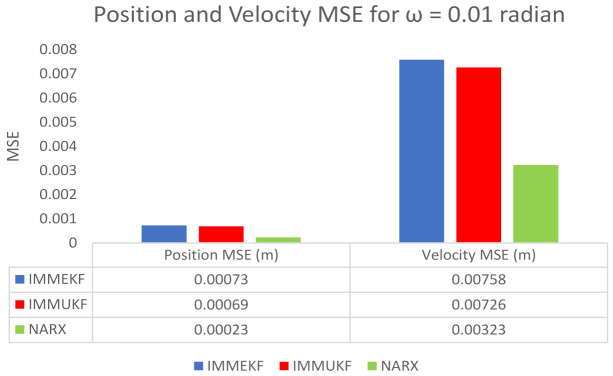
Average MSEs of predicting the position and velocity for IMMEKF, IMMUKF, and NARX in case 1.

**Figure 8:**
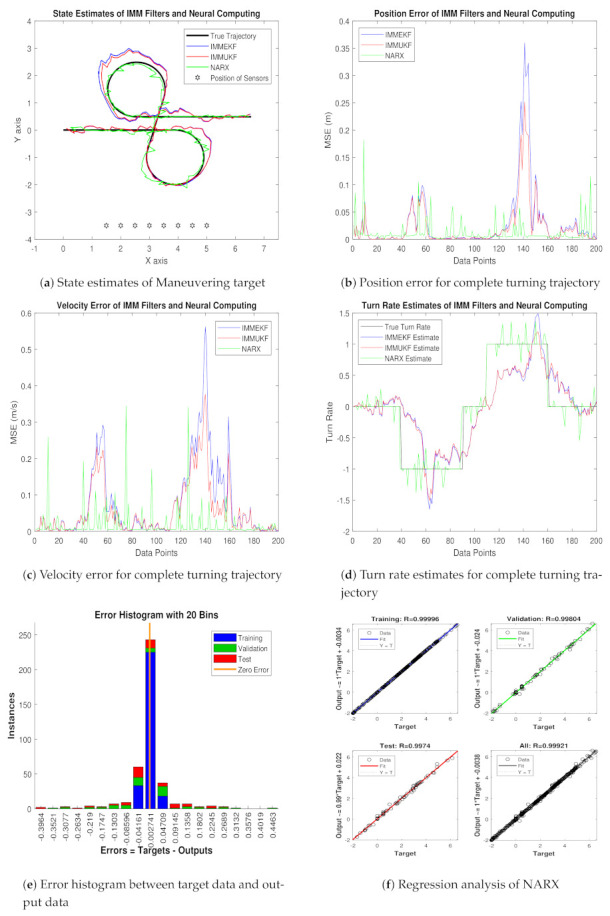
State estimation performance of IMMEKF, IMMUKF, and NARX for ω = 0.05 radian.

**Figure 9:**
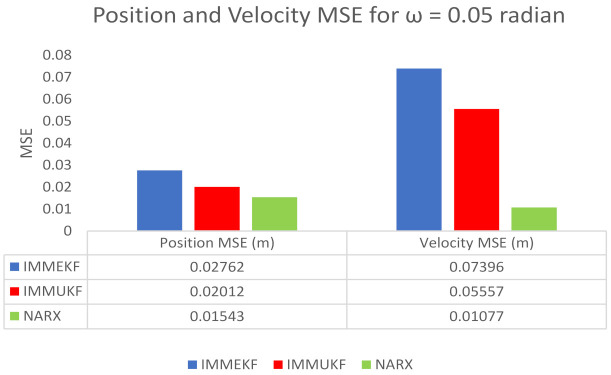
Average MSEs of predicting the position and velocity for IMMEKF, IMMUKF, and NARX in case 2.

**Figure 10:**
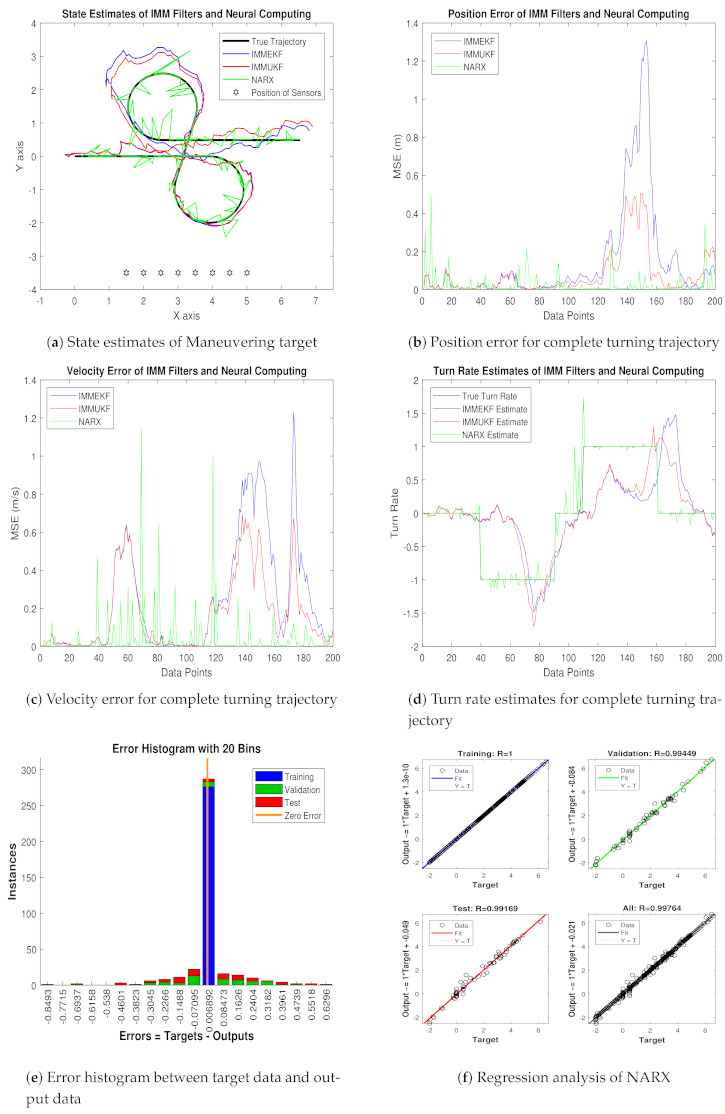
State estimation performance of IMMEKF, IMMUKF, and NARX for ω = 0.1 radian.

**Figure 11:**
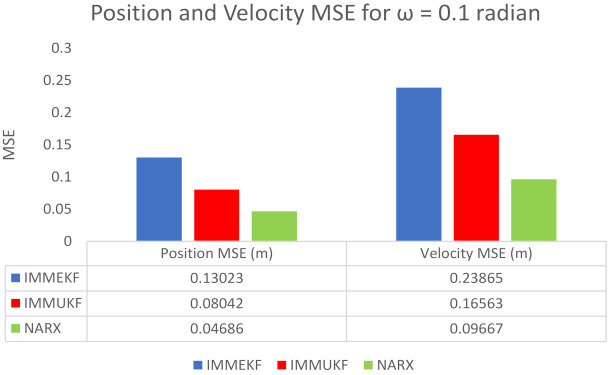
Average MSEs of predicting the position and velocity for IMMEKF, IMMUKF and NARX in case 3.

**Figure 12:**
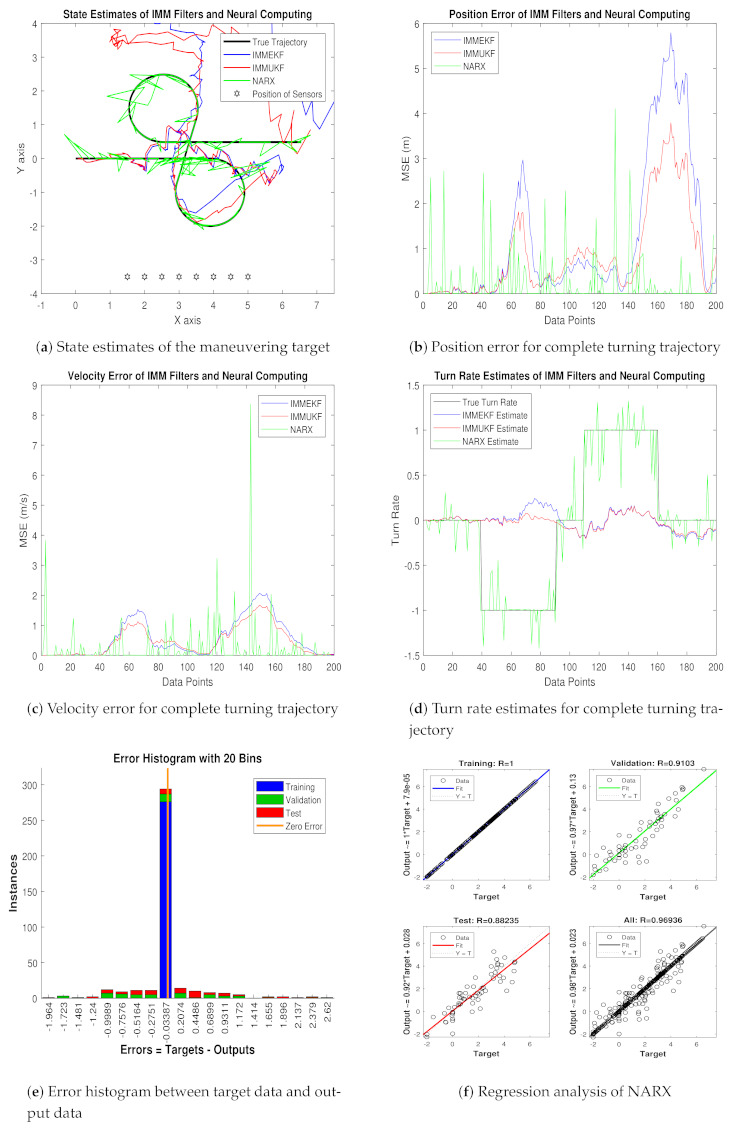
State estimation performance of IMMEKF, IMMUKF, and NARX for ω = 0.5 radian.

**Figure 13:**
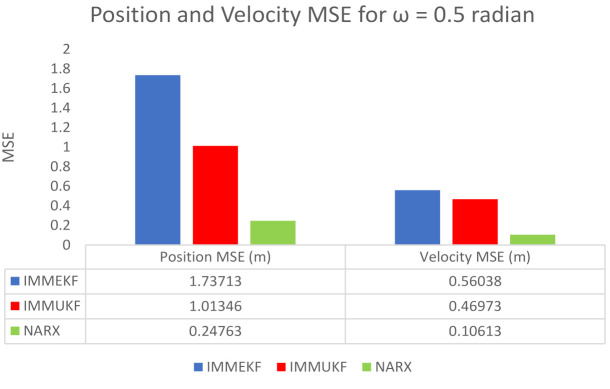
Average MSEs of predicting the position and velocity for IMMEKF, IMMUKF, and NARX in case 4.

**Figure 14:**
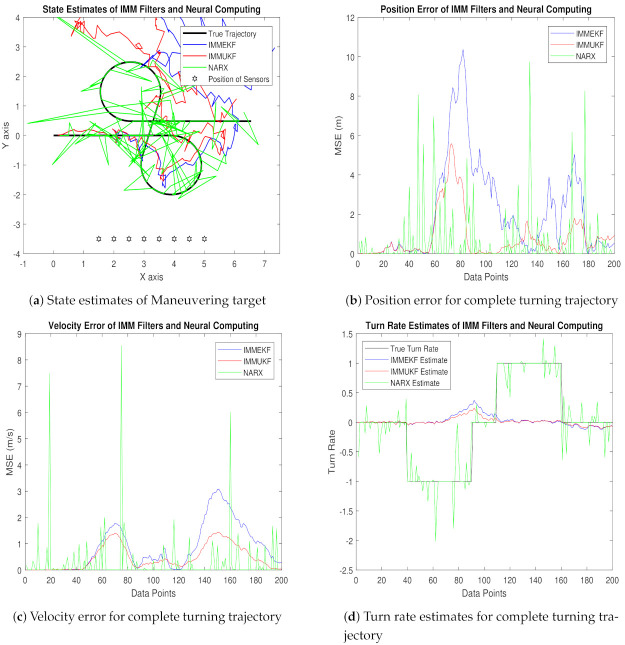

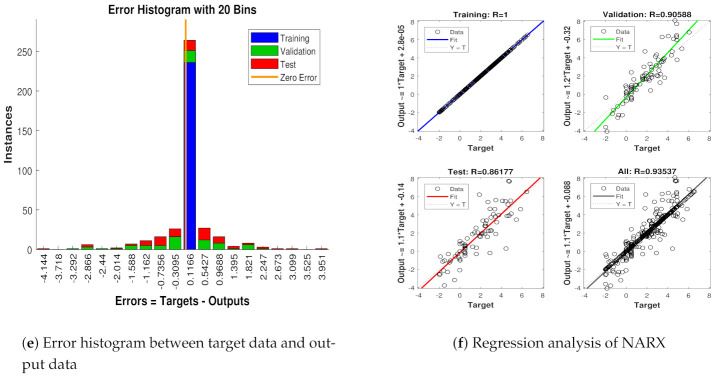
State estimation performance of IMMEKF, IMMUKF, and NARX for ω = 1 radian.

**Figure 15:**
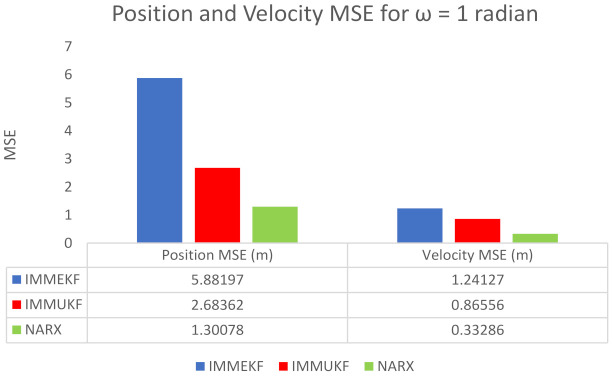
Average MSEs of predicting the position and velocity for IMMEKF, IMMUKF, and NARX in case 2.

In this subsection, MATLAB simulations’ results and their brief discussion are explained for real-time state prediction of the maneuvering object by applying IMMEKF, IMMUKF, and NARX neural intelligence computing. Accuracy and convergence based performance analysis of NARX is compared with IMM filters for five particular variations of the standard deviation of measured noise. In every case, state estimates, position error, velocity error, turn rate estimates, error histogram and regression analysis of GPB algorithms and NARX neural computing are presented from Figure 6, Figure 7, Figure 8, Figure 9, Figure 10, Figure 11, Figure 12, Figure 13, Figure 14 and Figure 15. These five different cases are discussed below with their mathematical expressions and simulation outcomes.

#### 4.1.1. Case 1: The Standard Deviation of Measurement Noise = 0.01 Radian

In the first case, the standard deviation of measured Gaussian noise ω is selected as a 0.01 radian that is depicting nearly an ideal and smooth underwater environment. Although covariance in this case is computed from the standard deviation of measurement noise as:

(37) $$T_{t}=diag(\omega_{X}^{2}),$$

while measurement noise at time instant t for e sensors is calculated from covariance as:

(38) $${\xi_{t}^{e}}^{M}\approx N\left(0,T_{t}\right).$$

For each passive bearing at time step t for sensor e, above the computed measurement noise, is integrating with measurement model X as:

(39) $$X_{t}^{e}=\mathrm{arctangent}\left[\frac{y_{t}-\Lambda_{y}^{e}}{x_{t}-\Lambda_{x}^{e}}\right]+{\xi_{t}^{e}}^{M}.$$

The above designed measurement model $X_{t}^{e}$ is applied at one input of the NARX neural model as input time series data. Another input of the NARX model is the target’s time series where the true state vector defined below is applied for obtaining an estimated state of underwater maneuvering target:

(40) $$Y_{t}=Y_{t}^{a}-Y_{t}^{b}={\left[\begin{array}{ccccc} x_{t} & y_{t} & {\dot{x}}_{t} & {\dot{y}}_{t} & \varphi_{t} \end{array}\right]}^{T}.$$

For this value of the standard deviation of measured noise in case 1, real-time state estimates, average true and estimated position and velocity error, true and estimated turn rate, error histogram, and regression analysis of real and predicted turning path are given as:

- In Figure 6a, a state estimation performance of NARX for a tracking turning trajectory of the maneuvering object is equated with GPB algorithms based on IMMEKF and IMMUKF, and it is quite vibrant that NARX is accurately tracing the actual turning path of the maneuvering target.
- Position error analysis in mean square sense is presented in Figure 6b in which NARX is showing the least error among other techniques.
- In Figure 6c, the error between true velocity and estimated velocity that is also presented in the context of MSE is shown. In this result, the NARX neural scheme is efficiently estimating true velocity compared to multiple model Kalman filters for all data points.
- Turn rate estimates of IMMEKF, IMMUKF, and NARX are illustrated in Figure 6d. Estimates of turning parameter are also validating the effectiveness of NARX over IMM filters for all data points in complete turning trajectory.
- Error histogram between target time series Y(t−1),Y(t−1),⋯,Y(t−n), and estimated output data $Y_{t}^{Est}$ are illustrated in Figure 6e. An error histogram consists of a set of error values that can be negative, and these define how much difference there is between estimated and target time series. In this analysis, the overall error of the NARX neuro network is divided into 20 bins that are shown in vertical bars. At the start of the histogram, a bin is unique from others having an error of 0.00017 at the height of 350 instances indicating that several points in time series have errors in this range. The zero error bar also falls on this bin which defines the zero error of the neural network.
- Regression analysis of the NARX scheme for training, validation, and testing procedure is shown in Figure 6f. The time series data set in the designed neural network is distributed in training, validation, and testing with the proportion of 75%, 15%, and 15%, respectively. This analysis integrates the statistical parameters for showing the relationship between the output variable $Y_{t}^{Est}$ and target variable Y(t). Here, in regression results, the true target and estimated output overlay each other. A linear trend is observed in both values that show the strength of NARX neural computing.

We also compute position and velocity MSE between real and estimated position and velocity of the object. These position and velocity error results also satisfy the graphical simulations that the precision of NARX is better from IMM Kalman filters. Position and velocity errors calculated for IMMEKF, IMMUKF, and NARX are enlisted in Figure 7.

#### 4.1.2. Case 2: The Standard Deviation of Measurement Noise = 0.05 Radian

The standard deviation of measurement noise is chosen as ω = 0.05 radian in case 2 for incorporating some amount of measurement noise in the estimation process. Meanwhile, covariance from this standard deviation of measurement noise is computed as:

(41) $$T_{t}=diag(\omega_{X}^{2}),$$

Gaussian distributed measurement noise at time step t for sensor e is depending upon above calculated covariance as:

(42) $${\xi_{t}^{e}}^{M}\approx N\left(0,T_{t}\right).$$

At each time instant t, measurement noise is including in the measurement model for sensor e as:

(43) $$X_{t}^{e}=\mathrm{arctangent}\left[\frac{y_{t}-\Lambda_{y}^{e}}{x_{t}-\Lambda_{x}^{e}}\right]+{\xi_{t}^{e}}^{M}.$$

Likewise, this designed measurement model $X_{t}^{e}$ is applied to the neural network as an input time series. At other inputs of NARX, the following state vector is applied as a target time series:

(44) $$Y_{t}=Y_{t}^{a}-Y_{t}^{b}={\left[\begin{array}{ccccc} x_{t} & y_{t} & {\dot{x}}_{t} & {\dot{y}}_{t} & \varphi_{t} \end{array}\right]}^{T}.$$

Real-time position of maneuvering object $\left(x_{t},y_{t}\right)$, velocity $\left({\dot{x}}_{t},{\dot{y}}_{t}\right)$, and turn rate $\varphi_{t}$ are actually the motion parameters which we want to estimate with NARX intelligence computing. Simulation results consisting of state estimates, position error, velocity error, turn rate estimates, error histogram, and regression analysis for this case are shown below:

- In Figure 8a, state estimates of all three techniques are compared for the coordinated turning trajectory of the maneuvering target. It is worth to note that, for this value of the standard deviation of measured noise, NARX is showing better state estimates and convergence than conventional nonlinear filtering methods.
- Average mean square position error among actual and predicted position of underwater maneuvering target is depicted in Figure 8b, which is representing the precision of NARX over IMMEKF and IMMUKF.
- In Figure 8c, true and estimated velocity of the maneuvering object estimated from IMM filters and NARX neural network is compared, and intelligence neural methodology is performing far better from filtering techniques.
- Figure 8d is showing turn rate estimates for this case of measurement noise in which again NARX is showing better estimates of turning parameter than existing filtering schemes.
- In Figure 8e, error histogram results are shown among target time series data set Y(t−1),Y(t−1),⋯⋯,Y(t−n), and predicted output value $Y_{t}^{Est}$ of the object’s state. In the middle part of histogram, a vertical bin consists of an error of 0.002741, and the length of this for the training dataset lies around 250 instances, while the validation and testing dataset lie between 200 and 250 instances. In this case, zero error lies beneath the vertical bar with center 0.002741.
- In Figure 8f, the regression of NARX neural intelligence computing is given for training, validation, and testing procedure. In regression analysis, the effectiveness of the NARX neural network is depicted by linear behavior and the head-to-head response of the true target and estimated output.

Position and velocity MSEs between actual and predicted position and velocity of turning target are computed for this variation of measurement noise. These position and velocity errors verify the previous results that estimation performance of NARX is higher than IMM Kalman filters, and it proves the strength of the neural methodology in state prediction of the maneuvering object. These position and velocity errors computed from IMMEKF, IMMUKF, and NARX are enlisted in Figure 9.

#### 4.1.3. Case 3: The Standard Deviation of Measurement Noise = 0.1 Radian

The standard deviation of measured Gaussian noise is increased to ω = 0.1 radian in this case representing that there is enough noise added in the system. The covariance T at time instant t from 0.1 radian standard deviation of Gaussian measurement noise is calculated as:

(45) $$T_{t}=diag(\omega_{X}^{2}),$$

Measurement noise from the above developed covariance for e sensor at time step t is defined as:

(46) $${\xi_{t}^{e}}^{M}\approx N\left(0,T_{t}\right).$$

Now, this generated amount of Gaussian measured noise is incorporating with the system model as:

(47) $$X_{t}^{e}=\mathrm{arctangent}\left[\frac{y_{t}-\Lambda_{y}^{e}}{x_{t}-\Lambda_{x}^{e}}\right]+{\xi_{t}^{e}}^{M}.$$

Measurement model X at time step t for sensor e is given in the above expression where passive bearings from all installed acoustic sensors are merging with white Gaussian distributed measurement noise. This measurement model expression is applied to the neural network model as an input dataset, while another input of the neural network consists of a given state vector of motion parameters:

(48) $$Y_{t}=Y_{t}^{a}-Y_{t}^{b}={\left[\begin{array}{ccccc} x_{t} & y_{t} & {\dot{x}}_{t} & {\dot{y}}_{t} & \varphi_{t} \end{array}\right]}^{T}.$$

Simulation results for this variation of measurement noise in the shape of state estimates, least position error, velocity error, turning parameter estimates, error histogram, and regression analysis are shown below:

- State prediction results in coordinated turn trajectory for all methods are given in Figure 10a, where the NARX based neural intelligence computing technique is showing better accuracy over conventional IMMEKF and IMMUKF. It can be seen that multimodel filters are experiencing more difficulty to estimate the state of the maneuvering object at turns of the trajectory than NARX, which is depicting the competency of the neural paradigm.
- Real-time positions errors of IMM filters and NARX are shown in Figure 10b in the context of the mean square. In this result, the accuracy of NARX is also better from other methods for all samples of turning trajectory.
- In Figure 10c, the velocity error of maneuvering object from all algorithms is shown in meter per second for all data points. The middle of the trajectory performance of NARX is not satisfactory, but, at the start and at the end of data points, NARX shows better performance than IMM filters.
- Turn rate estimates of all algorithms are presented in Figure 10d in which the estimation performance of neural computing is far better from Kalman filters.
- A comparative analysis among target time series Y(t−1),Y(t−1),⋯,Y(t−n), and the estimated value $Y_{t}^{Est}$ of target’s dynamics in the form of the histogram is represented in Figure 10e. A vertical bar incorporating an error of 0.006892 is shown in the center of the histogram. The height of this vertical bin for the training process is near 300 instances, whereas validation and testing dataset appears between 250 and 300 instances. Zero error for this analysis lies under the vertical bin with a value of 0.006892.
- Regression phenomena in graphical form for the procedures of training, validation, and testing are explained in Figure 10f. In this graph, a minimum divergence between target and output value is noted, which is because of an increment in the standard deviation of measured noise.

MSEs of position and velocity in meters and m/sec correspondingly between actual and predicted position and velocity of the target are also computed in this case. These position and velocity errors also approve the graphical results that precision of the neural network is far better with respect to multimodel filters. These position and velocity errors computed from IMMEKF, IMMUKF, and NARX are represented in Figure 11.

#### 4.1.4. Case 4: The Standard Deviation of Measurement Noise = 0.5 Radian

In case 4, the numerical value of measured noise is increased to ω = 0.5 radian, which is representing a sufficient quantity of Gaussian noise that is incorporated in the state estimation system. The mathematical expression of covariance designed with this measured noise is expressed as:

(49) $$T_{t}=diag(\omega_{X}^{2}),$$

while measurement Gaussian noise is computed from covariance as:

(50) $${\xi_{t}^{e}}^{M}\approx N\left(0,T_{t}\right).$$

Measured noise at times step t for e sensor is added in the measurement equation of the system as:

(51) $$X_{t}^{e}=\mathrm{arctangent}\left[\frac{y_{t}-\Lambda_{y}^{e}}{x_{t}-\Lambda_{x}^{e}}\right]+{\xi_{t}^{e}}^{M}.$$

The complete measurement model equation involving passive bearings and measurement noise is given to one input of the NARX neural model as an input time series $X_{t}^{e}$, while another input called target time series is the actual state vector is given below:

(52) $$Y_{t}=Y_{t}^{a}-Y_{t}^{b}={\left[\begin{array}{ccccc} x_{t} & y_{t} & {\dot{x}}_{t} & {\dot{y}}_{t} & \varphi_{t} \end{array}\right]}^{T}.$$

These real-time positions, velocities, and turn rates of kinetic passive maneuvering objects are developed for the absolute turning path of underwater targets and given in NARX neural design as target data for predicting these required motion parameters. In Figure 12, simulation results of state estimates, position error, velocity error, turn rate estimates, error histogram, and regression are shown as:

- In Figure 12a, state estimates of IMMEKF, IMMUKF, and NARX are presented in which the amount of measurement noise is high so all algorithms are experiencing difficulties to follow the real trajectory. However, even in the noisy atmosphere, it is seen that estimates of NARX are approaching a real trajectory more than the two other given algorithms.
- Average MSE among actual and predicted position of the maneuvering object is represented in Figure 12b, which is also showing that all algorithms have enough amount of error, but from a comparative point of view, NARX is estimating turning trajectory with less position error than IMMEKF and IMMUKF.
- In Figure 12c, velocity error from all methodologies is shown, which is also validating the strength of the neural network.
- Turn rate estimates in this case are presented in Figure 12d in which the estimation performance of neural computing is also better from IMM filtering methods.
- An error histogram among target values Y(t−1),Y(t−1),⋯⋯,Y(t−n), and output estimated value $Y_{t}^{Est}$ of target’s state is shown in Figure 12e. An error value −0.03387 in a vertical bin appears at the center of the histogram which has a height of close to 300 data points for the training dataset, while validation and testing datasets fall among 250 and 300 data sets. The zero error line on the error axis lies beneath the vertical with center −0.03387 in the given error histogram.
- Regression in this case is given in Figure 12f which is showing a sufficient amount of divergence between the true target and estimated output because of the increase in the standard deviation of measured noise.

The position and velocity MSEs between actual and predicted position and velocity of the maneuvering target are also calculated in this case. These position and velocity error responses also verify previous results that NARX is showing better performance from Kalman filters. These position and velocity errors computed from IMMEKF, IMMUKF, and NARX are defined in Figure 13.

#### 4.1.5. Case 5: The Standard Deviation of Measurement Noise = 1 Radian

In the last case of this study, we take a maximum value ω = 1 radian for assuming an extremely noisy atmosphere, and the state estimation system is facing a noisy measurement model. For this maximum value of ω, covariance $T_{t}$ is defined as:

(53) $$T_{t}=diag(\omega_{X}^{2}),$$

At time instant t, Gaussian distributed measurement noise ${\xi_{t}^{e}}^{M}$ is computed for e sensor from covariance $T_{t}$ as:

(54) $${\xi_{t}^{e}}^{M}\approx N\left(0,T_{t}\right).$$

Finally, this extreme value of independent white Gaussian measurement noise is included in measurement expression as:

(55) $$X_{t}^{e}=\mathrm{arctangent}\left[\frac{y_{t}-\Lambda_{y}^{e}}{x_{t}-\Lambda_{x}^{e}}\right]+{\xi_{t}^{e}}^{M}.$$

These passive measurements incorporated with maximum measurement noise are used as input time series $X_{t}^{e}$ of NARX neural architecture, while the target time series of the neural network is taken from an actual state vector, which is defined below:

(56) $$Y_{t}=Y_{t}^{a}-Y_{t}^{b}={\left[\begin{array}{ccccc} x_{t} & y_{t} & {\dot{x}}_{t} & {\dot{y}}_{t} & \varphi_{t} \end{array}\right]}^{T}.$$

The NARX based neural network is combining input time series and target time series to efficiently estimate output time series, which consist of the predicted state vector. In this case, results of state estimates, position error, velocity error, turn rate estimates, error histogram, and regression are shown in Figure 14.

- State estimates of IMMEKF, IMMUKF, and NARX in this case are presented in Figure 14a. State estimation results from all algorithms are diverging because of an extremely noisy underwater environment. All algorithms are facing severe difficulties in tracking true trajectory, but, in comparison, neural computing methodology NARX is performing better to estimate turning trajectory than the other two algorithms in this case.
- Average real-time error among the actual and predicted position of underwater maneuvering object is represented in Figure 14b in the form of MSE, which is clearly indicating that the NARX neural network is producing less position error as compared to IMMEKF and IMMUKF.
- In Figure 14c, velocity error results from all techniques are shown in which NARX is experiencing some large peaks but overall has better performance from IMM filters.
- Turn rate estimates in this case are representing in Figure 14d in which NARX is better estimating the turning parameter.
- An analysis of neural network in the form of error histogram is represented in Figure 14e among target dataset Y(t−1),Y(t−1),⋯⋯,Y(t−n), and predicted output $Y_{t}^{Est}$. A vertical bin is observed having an error of 0.1166 with a height near 250 steps for training while validation and testing datasets have a height between 200 and 250 steps. The zero error point lies at 0.1166 beneath the vertical bin.
- The regression of the NARX neural network between target and estimated output in this case is represented in Figure 14f. In regression results, a sufficient divergence is observed among the target and estimated results. This is because of a higher value of Gaussian noise involved in the state estimation system.

An extreme noisy environment position and velocity MSEs between actual and predicted position and velocity of the target are calculated in meters and m/s, respectively. These position and velocity error results confirm previous results that the accuracy of NARX is better from Kalman filters even in an extremely noisy underwater environment. Position and velocity errors computed from IMMEKF, IMMUKF, and NARX are enlisted in Figure 15.

The above figures of all simulation results prove that all state prediction methods are facing problems with following the real state of turning maneuvering objects at higher arithmetic values of measurement noise ω in an underwater atmosphere. While in a comparative point of view among all methods, NARX based neural intelligence computing is performing far better, which is proving its effectiveness for nonlinear real-time state prediction applications in the underwater scenario.

## 5. Conclusions

In this paper, we investigated the neural intelligence computing paradigm based on NARX for real-time state estimation of a bearings only maneuvering Markov chain underwater object. At each time instant, the instantaneous positions of a continuously turning object are estimated in the bi-dimensional Cartesian geometric system. Initially, the state space-based estimation framework for a dynamic and measurement model using BOT phenomena are designed mathematically. Then, the neural computing technique based on NARX is designed for efficient state estimation of a passive maneuvering Markov chain target. In this context, the NARX neural strategy is examined in MATLAB for 200 data points, and we assessed the robustness of neural computation for an absolute turning trajectory of object motion in the sense of turn rate estimation, current position estimation, real-time state estimation, and velocity error along with error histogram and regression. Later, enough variations of standard deviation of measurement Gaussian noise are used for examining the performance of the designed method. State estimation results endorsed the competency of the NARX neural technique as compared with typical nonlinear multiple model Bayesian filtering methods like IMMEKF and IMMUKF. However, an exponential decay in the simulation results of all methods is observed in a noisy underwater scenario. Therefore, determining precise state prediction performance in a cluttered sea atmosphere is still a challenge.

In the future, this work can be extended by investigating the radial base function (RBF) neural methodology for getting more accurate state estimation of a constant velocity multiple model turning single or multi objects by applying non-Gaussian noise [45,46], which is an interesting research domain in the field of acoustic signal processing. In addition, in future studies, a detailed complexity analysis can be conducted for analyzing time delay between NARX neural computing and filtering strategy.

## Figures and Tables

**Table 1:** Appropriate setting of different state estimation variables.

Parameters	Appropriate Values
Starting state of the target	$Y_{0}$ = ${\left[0\;0\;1\;0\;0\right]}^{T}$
Localization function of sensors	$\left(\Lambda_{x}^{e},\Lambda_{y}^{e}\right)$
Total sensors	*e* = 8
Distance between sensors	0.5
Values of Standard deviation of measured noise	ω=0.01→1 radians
Process noise variance of WPV model	$b_{1}$ = 0.05
Process noise variance of CT model	$Y_{\varphi }$ = 0.15
Sampling space	Δt= 0.1 s
Input and output delays	m, n = 2
Total sample points	200
Hidden neurons	100
NARX target steps	1000

## Data Availability

Not applicable.
